# Neglected and Emerging Tropical Diseases in South and Southeast Asia and Northern Australia

**DOI:** 10.3390/tropicalmed3030070

**Published:** 2018-06-22

**Authors:** Peter A. Leggat, Patricia Graves, Thewarach Laha, Khin Saw Aye

**Affiliations:** 1 World Health Organization Collaborating Centre for Vectorborne and Neglected Tropical Diseases, College of Public Health, Medical and Veterinary Sciences, James Cook University, Townsville, QLD 4811, Australia; patricia.graves@jcu.edu.au; 2 Faculty of Science, University of Nottingham Malaysia Campus, Jalan Broga, Semenyih 43500, Selangor Darul Ehsan, Malaysia; 3 Department of Parasitology, Faculty of Medicine, Khon Kaen University, Khon Kaen 40002, Thailand; thewa_la@kku.ac.th; 4 Department of Medical Research, Ministry of Health and Sports, Yangon 11191, Myanmar; ksadmr@gmail.com

This Special Issue focuses on recent research on the important emerging and neglected tropical diseases (NTDs) in South and South East Asia and Northern Australia. This region stretches from Pakistan in the west to the Philippines in the east, and includes Afghanistan and countries to the east, the Indian subcontinent, mainland South-East Asia, and the tropical regions of Australia. Many of these areas are highly endemic for important NTDs and other tropical diseases, including lymphatic filariasis (LF), soil-transmitted helminthiases (STH) such as hookworm infection, trichuriasis, ascariasis, and strongyloidiasis, rickettsial diseases and arboviral diseases. Several of these diseases are targeted for elimination or enhanced control by the World Health Organization (WHO) in the next 5 to 10 years, although some have chronic lasting sequelae and disability needing lifelong management. Control methods used include preventive chemotherapy, enhanced screening and treatment, intensified disease management, vector control, interruption of human to animal transmission, environmental/sanitation improvements and disability prevention/mitigation. A current list of WHO NTDs is given in Table 1. 

At the time of publication, there have been 11 papers published upon peer review acceptance in this Special Issue, including eight original papers, two review papers and one perspectives piece. They each contribute to a much better understanding of Neglected and Emerging Tropical Diseases in South and Southeast Asia and Northern Australia. The contributions to these topics can be summarized as follows: four submissions on LFs [2,3,4,5], four submissions on STHs [6,7,8,9], two submissions on rickettsial diseases [10,11], and one submission on arboviral diseases [12]. A systematic review and meta-analysis leads the opening section on LF [2], which reviews prevalence and disease burden of LF in southeast Asia [2]. Two studies in Myanmar review the utility of dried blood spots on filter paper for sampling for detection Bm14 antibody and Og4C3 antigen in cases of LF [3,4], with the latter indicating need for reconciliation between different sampling methods. A further study in Myanmar examined the usefulness of low-cost devices for measuring tissue compressibility and extracellular fluid, used and accepted in other clinical settings, for objective assessment of lymphedema [5]. A review paper leads the other major section on STH, which focuses on the prevalence of STHs in different groups, including immigrants, travellers, military personnel and veterans in Australia and Asia [6]. This is followed by studies examining an extended period of surveillance data on *Strongyloides stercoralis* [7]; and a study examining the prevalence of STHs in remote Aboriginal communities, both in the Northern Territory, Australia [8]; and a study examining the links between dietary intake, nutritional status, and intestinal parasites, such as *Schistosoma japonicum*, *Ascaris lumbricoides*, *Trichuris trichiura*, and hookworm, in the Philippines [9]. The two rickettsial papers examine hospital admissions for Queensland tick typhus in north Brisbane, Australia [10], and the other study based in Thailand looks at the influence of land use on scrub typhus in rodents [11]. Lastly, a perspective piece reminds us that Australia is home to more than 75 arboviral diseases-, which pose a public health threat to the Australian population [12].

The diversity of papers, the depth of the topics and the relative geographical reach of the authors (including authors from several countries across Asia, as well as authors from Australia and Europe) in this Special Issue confirm the continued collective major interest in this area. This wide-ranging open access collection contributes to a much better understanding on the epidemiology, presentation, diagnosis, treatment, prevention and control of neglected and emerging tropical diseases in South and Southeast Asia and Northern Australia. As the editors of this Special Issue, we trust that you find the content useful, as the authors are pleased to share their knowledge with an international audience. We look forward to future opportunities to update advances in this field and encourage you or publish your work in or propose a Special Issue for *Tropical Medicine and Infectious Disease*.

## Figures and Tables

**Table 1:** Neglected Tropical Diseases [1].

Neglected Tropical Diseases
Buruli ulcer
Chagas disease
Dengue and Chikungunya
Dracunculiasis (guinea-worm disease)
Echinococcosis
Foodborne trematodiases
Human African trypanosomiasis (sleeping sickness)
Leishmaniasis
Leprosy (Hansen’s disease)
Lymphatic filariasis
Mycetoma, chromoblastomycosis and other deep mycoses
Onchocerciasis (river blindness)
Rabies
Scabies and other ectoparasites
Schistosomiasis
Soil-transmitted helminthiases
Snakebite envenoming
Trachoma
Yaws (Endemic treponematoses)
Taeniasis/Cysticercosis
